# Replication of porcine circoviruses

**DOI:** 10.1186/1743-422X-6-60

**Published:** 2009-05-18

**Authors:** Florence Faurez, Daniel Dory, Béatrice Grasland, André Jestin

**Affiliations:** 1 French Food Safety Agency (Afssa), Viral Genetics and Biosafety Unit, Fr-22440, Ploufragan, France

## Abstract

Porcine circoviruses are circular single-stranded DNA viruses that infect swine and wild boars. Two species of porcine circoviruses exist. Porcine circovirus type 1 is non pathogenic contrary to porcine circovirus type 2 which is associated with the disease known as Post-weaning Multisystemic Wasting Syndrome. Porcine circovirus DNA has been shown to replicate by a rolling circle mechanism. Other studies have revealed similar mechanisms of rolling-circle replication in plasmids and single-stranded viruses such as *Geminivirus*. Three elements are important in rolling-circle replication: i) a gene encoding initiator protein, ii) a double strand origin, and iii) a single strand origin. However, differences exist between viruses and plasmids and between viruses. Porcine circovirus replication probably involves a "melting pot" rather than "cruciform" rolling-circle mechanism.

This review provides a summary of current knowledge of replication in porcine circoviruses as models of the *Circovirus *genus. Based on various studies, the factors affecting replication are defined and the mechanisms involved in the different phases of replication are described or proposed.

## Introduction

**Figure 1:**
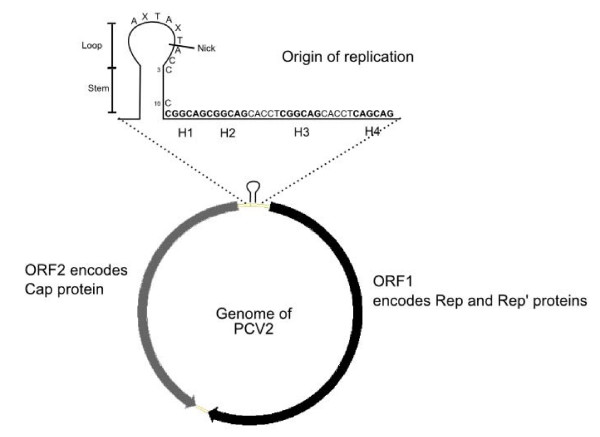
**Representation of genome of PCV-2**. Black arrow: Open Reading Frame 1 (ORF1), located on the positive strand, which encodes Rep and Rep' protein. Grey arrow: Open Reading Frame 2 (ORF2), located on the negative strand, which encodes Cap protein. Between ORF1 and ORF2 are intergenic regions. The origin of replication is located in the intergenic region between the beginnings of the two ORFs. H1, H2, H3 and H4 are hexamers.

The members of the *Circovirus *genus in the *Circoviridae *family are animal viruses, most of which affect birds although type 1 and type 2 porcine circoviruses (PCV-1 and PCV-2) affect swine and wild boars. All circoviruses known at present, except PCV-1, are associated with immuno-suppressive or immuno-depressive diseases. They have small (around 2 kb), closed-circular, single-stranded (ss) DNA, ambisense genomes. The intergenic region contains the origin of replication with a stem loop structure which includes an octanucleotide sequence flanked by palindromes, and is bordered by two open reading frames, ORF1 and ORF2 (figure 1). ORF1 is located on the positive strand and encodes the Rep and Rep' proteins involved in replication initiation. ORF2 is located on the negative strand of the replicating double-stranded PCV-2 genome and encodes the Cap protein which composes the capsid of this virus. As porcine circoviruses (PCVs) are the most studied, they can be considered as models.

It is apparent from the nucleic acid sequence that the PCV genome might be at the interface of Geminivirus and Nanovirus genomes [1]. The Rep protein sequence suggests that the PCV genome results from a Nanovirus and Calicivirus recombination [2].

Based on common features which include the initiator Rep protein and stem-loop structure, it has been proposed that PCVs, like *Geminiviridae *and *Nanoviridae*, replicate using a rolling circle replication (RCR) strategy [3,4]. This mechanism is also involved in replication of the pT181 plasmid of Staphylococcus aureus which encodes resistance to tetracycline [5]. Studies of PCV replication have been based on the RCR model but a novel mechanism or "melting-pot RCR model" has been proposed which involves modifications at the origin of replication [6]. This review provides a synthesis of current knowledge of replication in PCVs as models of the *Circovirus *genus.

## General model of rolling-circle replication

**Figure 2:**
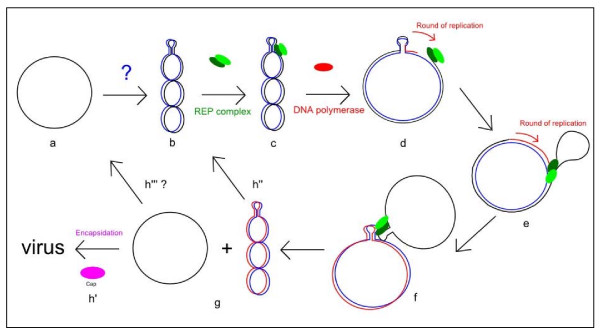
**Rolling circle replication model of single strand viruses**. Black strand: positive parental strand and viral genome. Blue strand: negative lagging strand. Red strand: positive leading strand. a and b: the conversion of the single strand viral genome in the double strand DNA replicative intermediate is produced in the cell. c and d: REP complex binds to the stem-loop structure which is the origin of replication and initiates the replication by nicking the DNA. d and e: cellular DNA polymerase initiates viral DNA replication from the free 3'OH extremity and the REP complex still binds the 5' extremity. f and g: after a round of replication, the REP complex closes the DNA and releases a single strand DNA and a double strand DNA. h', h" and h"': DNA can be used for replication or can be encapsidated.

Synthesis of the leading strand and the lagging strand is uncoupled and rolling-circle replication is both unidirectional and asymmetric. After the infection of permissive cells, synthesis of the lagging strand, involving a still-unknown mechanism in PCVs, starts from the viral positive single-stranded DNA. This produces the supercoiled double-stranded replicating form (RF) of the genome (figure 2b). Thereafter, a RCR initiator protein complex binds to the stem loop structure (figure 2c) and destabilizes the replication origin. This complex is composed of newly synthesized Rep and Rep' proteins in PCVs [7] or of homodimer Rep proteins in some plasmids [5] and Geminivirus [3]. The RCR initiator protein cleaves the loop to generate a free 3'OH extremity and covalent binding between the complex and the 5' viral genome extremity. Cellular DNA polymerase initiates viral DNA replication from the free 3'OH extremity (figure 2d). When 1 unit of the genome has been replicated, the REP complex closes the loop covalently. This leads to the release of a positive circular single-stranded parental DNA molecule and a circular double-stranded DNA molecule composed of the negative parental strand and the newly synthesized positive strand (figure 2f and 2g). The single-stranded DNA molecule will then either be encapsidated (figure 2h') or, as in Geminiviruses, involved in a second replication cycle (figure 2h"'). A 3 to 5 fold increase of double-stranded DNA was observed for Geminiviruses in the absence or inactivation of Cap proteins [8]. However, the mechanisms governing the regulation of these two possibilities are unknown.

## Replication factors

PCV replication studies have generally been performed on the PCV-1 model. Nevertheless, PCV-1 and PCV-2 exhibit 79.5% and 82% sequence homologies for the replication origin and Rep gene, respectively [9], and one replication factor can be replaced by another [10]. Therefore, hypotheses formulated for PCV-1 replication might probably be valid for PCV-2.

### Leading strand replication origin

**Figure 3:**
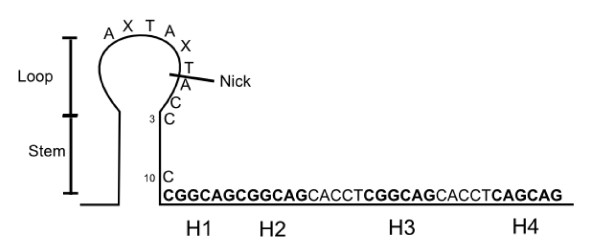
**PCV-2 replication origin**. H1: Hexamer 1; H2: Hexamer 2; H3: Hexamer 3; H4: Hexamer 4 Blue nucleic acids: pentamers CACCT.

The replication origin of the leading strand is a stem-loop structure. An AxTAxTAC sequence is located on the loop and the stem is a 10 nucleotides palindrome in which the 2 Cs at positions 3 and 10 of the right stem play a role in binding the REP complex to the DNA (Figure 3). Four hexanucleotide sequences (for PCV-2: H1, H2 and H3: CGGCAG and H4: CAGCAG) are present downstream from the stem structure. H1 and H2 constitute the Rep and Rep' *in vivo *proteins binding sites [11]. The stem-loop structure plays an important role in the termination of replication [12] whereas the octanucleotide sequences in the loop, H1 and H2, are necessary to its initiation. The replication origins of rolling circle replicative plasmids show structural similarities to those of the pT181 family [5], Nanoviruses [4] and Geminiviruses [3].

### REP Complex

Replication of the PCV genome, unlike that of plasmids and *Geminiviruses*, requires the REP complex which consists of 2 viral proteins Rep and Rep' [13]. The *rep *gene encoding for these 2 proteins is located on the positive strand downstream from the replication origin and its promoter includes the replication origin and to a certain extent ORF2. Rep consists of 314 amino acids and 37.5 kDa protein, whereas Rep', which is a spliced form of the Rep mRNA, consists of 178 amino acids and 20.2 kDa protein [10]. The C-term parts of Rep' and Rep are also different. The N-term portions of Rep and Rep' contain three conserved amino acid sequences typical of proteins initiating RCR [14,15].

The three-dimensional structures of the geminivirus Tomato Yellow Leaf Curl Sardinia Virus (TYLCSV) [16], in the nanovirus Faba Bean Necrotic Yellow Virus (FBNYV) [17] and in the circovirus PCV-2 [18] have been described. According to structural data, the three RCR motives in TYLCSV, FBNYV and PCV-2 are similarly positioned on the various elements of the Rep proteins structure.

**Figure 4:**
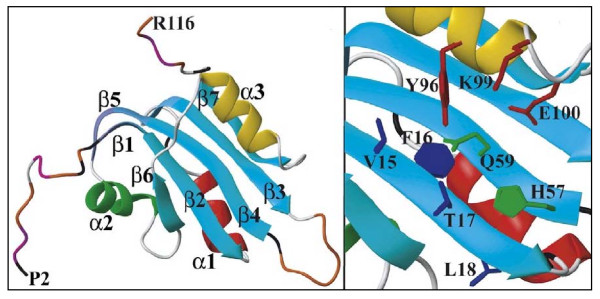
**3D structure of the Rep protein N-term part of PCV-2**. Left panel: Gibbon representation. In blue: 5 β sheets; red and green: α helix; grey: loop; yellow: α3 helix bearing the catalytic site. Right panel: zoom on β sheets and catalytic α helix. Blue amino acids: motif I (FTLN) of β2 sheet; Green amino acids: motif II (HLQGF) of β4 sheet; Red amino acids: motif III (YCSK). Reprinted from Journal of Molecular Biology, 367(2), Vega-Rocha S et al, "Solution structure, divalent metal and DNA binding of the endonuclease domain from the replication initiation protein from porcine circovirus 2.", pages 473–487, Copyright^© ^2007 [18], with permission from Elsevier.

The role of motif I in the Rep and Rep' proteins of PCV-2 i.e., FTLN located on the β5 sheet [18], is unknown. However, motif II i.e., HxQ located on the β4 sheet [18], seems to play a role in the metallic binding which is essential to the initial cleavage. Motif III, an YxxK motif located on the α3 helix [18], contains the tyrosin which catalyzes DNA cleavage [19] (figure 4). A GKS box or P-loop, hydrolysing ATP in the presence of Mg^2+ ^ions [20] and a helicase domain [12], have been identified on the C-term portion of the Rep protein. The Rep protein therefore seems to play a helicase role, whereas the Rep' protein, being devoid of this kind of structure, may be involved in nuclease activity. The Rep protein negatively regulates its promoter whereas the Rep' and Cap proteins do not have any effect on this promoter [9]. This regulation requires the Rep elements which bind to hexamers H1 and H2, and also motifs I and II [21]. The C-term portion of Rep and Rep' could also play a role in transcriptional regulation by interaction with cellular proteins like ZNF265, VG5Q and TDG [22].

## Melting-pot RCR model

### Synthesis of the lagging strand

The first step in RCR is synthesis of the lagging strand which results in passage from single-strand DNA to the replicating double-strand DNA. As yet, neither the PCV genomic DNA sequence nor the proteins involved in this synthesis have been determined. However, the replication mechanisms described for RCR plasmids, Nanoviruses and Geminiviruses might provide some indication of the PCV mechanism.

In RCR plasmids, synthesis is initiated and terminated at a single-stranded origin. This origin is a non-coding region that can generate an imperfect stem-loop and is usually located away from the double-stranded origin involved in leading strand synthesis. The single-stranded origin contains a promoter recognized by a host RNA polymerase that synthesizes an RNA primer essential to DNA synthesis by cellular DNA polymerase I [5]. Two mechanisms have been described for different groups of Geminiviruses. Mastreviruses (subgroup I) encapsidate DNA molecules about 80 nucleotides in length that are complementary to a single-stranded Small Intergenic Region (SIR) of the virus genome. This small intergenic region is different from the intergenic region which contains the origin of replication. The complementary DNA molecule consists of ribonucleotides at the 5' end which induce synthesis of the lagging strand [23]. Such DNA molecules have not been detected for Curtoviruses (subgroup II) or Begomoviruses (subgroup III), except in the African cassava mosaic virus that belongs to subgroup III [24]. The origin of the negative strand of Curtoviruses and Begomoviruses seems to be located in the intergenic region which contains the origin of replication and, contrary to Mastreviruses, a RNA primer is probably synthesized after infection [3]. The presence of a DNA molecule with the same function as in Mastreviruses was also described for one Nanovirus but no ribonucleotide was identified [25].

The origin of replication of the lagging strand in PCV has not yet been identified. However the Geminivirus and Nanovirus models might help to explain PCV replication. It is possible that the origin of synthesis of the negative strand of the PCV genome is located in the intergenic region, which may or may not contain the origin of replication. A DNA molecule might thus either be encapsidated like Mastrevirus or produced after infection like Begomovirus.

### Leading strand synthesis: initiation

Initiation of the leading strand first requires binding of the REP complex to the replicating form of the PCV genome, followed by nicking of the DNA by this complex.

The replication origin contains the elements essential to REP complex binding. The Rep protein binds to hexamers H1 and H2 and to the right strand of the stem-loop whereas the Rep' protein binds only to hexamers H1 and H2 [11]. Rep protein binding requires cytosines at positions 3 and 10 of the right strand of the stem-loop motif (figure 3) and an appropriate spatial distance between these nucleotides [26]. Binding of Rep and/or Rep' to the viral genome might be influenced by other elements in the PCV genome such as the octanucleotide in the loop [27]. The binding interface of the Rep protein includes two structural elements. The first element is the C terminal of the α helix (α2 helix and α3 helix). The second element is the loop between the β4 sheet and the β5 sheet [18] (figure 4).

The replication origin is destabilized by the REP complex bound to the DNA. The REP complex nicks then the AxTAxTAC sequence of positive single stranded DNA (+) between nucleotides T and A (figure 3). The porcine circovirus cleavage mechanism presents similarities to that of Geminivirus. In both cases, cleavage is dependent on the presence of divalent cations such as Mg^2+^, but not of ATP or of the stem-loop structure [12,28]. In PCV, the catalytic site responsible for this cleavage is tyrosine 96 (Tyr96) located on motif III of the Rep protein. Cleavage of the phosphodiester bond usually occurs by nucleophilic attack of the hydroxyl group of tyrosine 96. This attack generates a free 3'OH end and a phosphotyrosine diester binding at the 5' site [20]. Replication of the positive viral DNA strand by the host DNA polymerase is initiated at the free 3'OH end. This 3'OH end is used as a primer. Several steps are needed to initiate replication: recruitment of the REP complex, cleavage and recruitment of the host DNA polymerase. Each step may interact with each of the others.

### Leading strand synthesis: initiation/elongation

**Figure 5:**
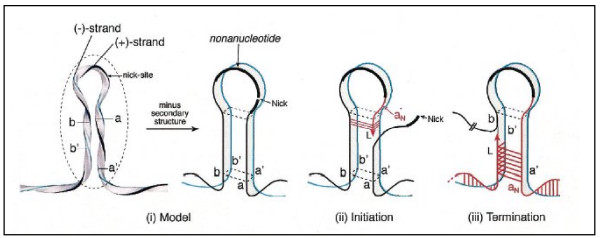
**Melting pot rolling circle replication model**. i. Replication origin representation after binding of the Rep. Strands (+) and (-) are close together. The destabilized environment known as "melting pot" is included in the dotted oval. ii. Schematic representation of the template DNA strands during the initiation of DNA synthesis. The leading strand shifts strand 'a', and uses strands 'a' and 'b' as template DNA strands. iii. Schematic representation of the template DNA strands at the termination of DNA synthesis. The leading strand shifts strand 'b' and uses either the newly synthesized 'a_N _' or strand 'b'. Black: positive polarized genome (+); Blue: negative strand; Red: potential base pairs representation. Reprinted from Journal of Virology **78**(8), Cheung AK, "Detection of template strand switching during initiation and termination of DNA replication of porcine circovirus." pages 4268–4277, Copyright^© ^2004 [6], with permission from American Society for Microbiology.

Unlike the cruciform rolling-circle model of the Geminiviruses, PCVs exhibit "melting pot" organization [6]. This is described as a sphere of instability at the level of the stem-loop where the (+) and (-) strains are close together but not held by hydrogen bonds. This allows a possible exchange of matrix strand(s), also called template strand(s), during genome synthesis (figure 5). The rolling circle melting pot model, as defined by Cheung [6], was thoroughly investigated by Kato's team in 2003 [29]. This study revealed the ability of a quadruplet structure to generate transient triplex stem loop configurations, thereby making the two template strands available for positive strand synthesis and allowing easy exchange of these template strands. The availability of two template strands during synthesis of the leading strand seems to help maintain the nucleic sequence of the replication origin.

### Synthesis of the leading strand: termination

**Figure 6:**
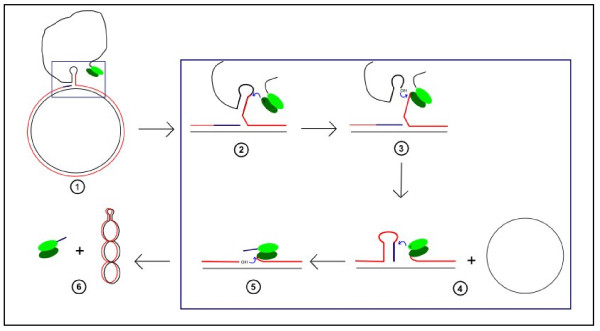
**Model of the termination of rolling circle replication described by Novick in the nineties**. Black: parental strand; Red: newly synthesized strand; Blue: newly synthesized strand during a second round of replication. Step 1: After the first round of replication, the REP complex bound on the 5' end would be situated behind the generated stem-loop. Step 2: the tyrosine (Y) of the unused sub-unit during the initiation might cleave the regenerated replication origin. It will (then bind to the 5' end of the newly synthesized strand. Step 3: the 3'OH free end generated during step 2 and belonging to the parental strand might then exert a nucleophilic attack on the tyrosylphosphodiester bridge generated during the initiation step. This reaction would lead to the release of a single stranded DNA. Step 4: the tyrosine previously involved in initiation of the replication attacks the cutting site of the newly synthesized strand. This reaction would generate a free 3'OH on the newly synthesized strand and a tyrosylphosphodiester bridge with a 12 mers oligonucleotide. Steps 5 and 6: the free 3'OH generated at step 4 can attack the tyrosylphosphodiester bridge generated at step 2, that would close the double stranded DNA and release an inactivated Rep homodimer due to the binding of one tyrosine to one oligonucleotide. Adapted from Microbiology and Molecular Biology Reviews **62**(2), del Solar G et al, "Replication and control of circular bacterial plasmids." pages 434–464, Copyright^© ^1998 [5], with permission from American Society for Microbiology.

A termination model of rolling circle replication based on pT181 plasmid studies was described in the nineties by Novick's team [30]. This mechanism is based on nucleophilic attacks on a tyrosylphosphodiester bond by a free 3'OH and on catalytic activity of a tyrosine on the DNA (figure 6). This leads to the release of a covalently closed circular single-strand DNA, a covalently closed circular double-strand DNA and an inactivated Rep heterodimer. The inactivated Rep heterodimer might result from binding of the tyrosylphosphodiester in a 12-mers oligonucleotide to one of the Rep proteins (figure 6, step 6). This oligonucleotide comes from the newly synthesized strand which is slightly more than one genome unit long (figure 6, step 4).

The termination mechanism of PCV leading strand synthesis has not yet been studied. However the involvement of tyrosine in PCV replication suggests that the mechanism might be similar to that of the pT181 plasmid.

## Conclusion

This mini-review summarizes the various mechanisms and elements necessary for rolling circle replication of porcine circovirus. Although not all these mechanisms have been clarified, the explanatory indications are based on sequence homology between porcine circovirus and other models such as Geminivirus or pT181 plasmids. Supplementary studies on porcine circovirus should validate or not the hypotheses associated with the various models.

## Competing interests

The authors declare that they have no competing interests.

## Authors' contributions

FF was the main author of this review. DD, BG and AJ read and completed the review. All authors read and approved the final manuscript.
